# Multicentre randomized controlled trial of angiotensin-converting enzyme inhibitor/angiotensin receptor blocker withdrawal in advanced renal disease: the STOP-ACEi trial

**DOI:** 10.1093/ndt/gfv346

**Published:** 2015-09-30

**Authors:** Sunil Bhandari, Natalie Ives, Elizabeth A. Brettell, Marie Valente, Paul Cockwell, Peter S. Topham, John G. Cleland, Arif Khwaja, Meguid El Nahas

**Affiliations:** 1 Department of Renal Medicine, Hull and East Yorkshire Hospitals NHS Trust, Kingston upon Hull, UK; 2 Hull York Medical School, East Yorkshire, UK; 3 Birmingham Clinical Trials Unit, University of Birmingham, Birmingham, UK; 4 Department of Renal Medicine, Queen Elizabeth Hospital, Birmingham, UK; 5 Department of Renal Medicine, Leicester General Hospital, Leicester, UK; 6 National Heart & Lung Institute, Imperial College London, London, UK; 7 Sheffield Kidney Institute, Sheffield, UK

**Keywords:** angiotensin-converting enzyme inhibitor (ACEi), angiotensin II receptor blocker (ARB), chronic kidney disease, eGFR, randomized controlled trial

## Abstract

**Background:**

Blood pressure (BP) control and reduction of urinary protein excretion using agents that block the renin–angiotensin aldosterone system are the mainstay of therapy for chronic kidney disease (CKD). Research has confirmed the benefits in mild CKD, but data on angiotensin-converting enzyme inhibitor (ACEi) or angiotensin receptor blocker (ARB) use in advanced CKD are lacking. In the STOP-ACEi trial, we aim to confirm preliminary findings which suggest that withdrawal of ACEi/ARB treatment can stabilize or even improve renal function in patients with advanced progressive CKD.

**Methods:**

The STOP-ACEi trial (trial registration: current controlled trials, ISRCTN62869767) is an investigator-led multicentre open-label, randomized controlled clinical trial of 410 participants with advanced (Stage 4 or 5) progressive CKD receiving ACEi, ARBs or both. Patients will be randomized in a 1:1 ratio to either discontinue ACEi, ARB or combination of both (experimental arm) or continue ACEi, ARB or combination of both (control arm). Patients will be followed up at 3 monthly intervals for 3 years. The primary outcome measure is eGFR at 3 years. Secondary outcome measures include the number of renal events, participant quality of life and physical functioning, hospitalization rates, BP and laboratory measures, including serum cystatin-C. Safety will be assessed to ensure that withdrawal of these treatments does not cause excess harm or increase mortality or cardiovascular events such as heart failure, myocardial infarction or stroke.

**Results:**

The rationale and trial design are presented here. The results of this trial will show whether discontinuation of ACEi/ARBs can improve or stabilize renal function in patients with advanced progressive CKD. It will show whether this simple intervention can improve laboratory and clinical outcomes, including progression to end-stage renal disease, without causing an increase in cardiovascular events.

## INTRODUCTION

Chronic kidney disease (CKD), Stages 3–5, affects 1 in 10 adults in the UK and reflects progressive scarring of the kidneys regardless of the original disease and is associated with a high prevalence of cardiovascular disease [1]. Advanced CKD (Stage 4 or 5) is associated with a significantly increased risk of death [hazard ratio (HR): 3.6; 95% confidence interval (CI) 3.2–4.0] [2] and a 50-fold increased requirement for dialysis, in comparison with age-matched individuals with presumed normal kidney function [3–6]. Advanced CKD has a major negative impact on a range of other clinical outcomes including quality of life [7, 8] and carries a high economic burden either through associated cardiovascular or metabolic bone disease or due to the high cost of renal dialysis (∼£30 000/year).

Irrespective of the underlying cause of CKD, attention has focused primarily on blood pressure (BP) control and reduction of urinary protein excretion using agents that block the renin–angiotensin aldosterone system and reduce intra-glomerular pressure over and above their effect on BP. Studies by Lewis *et al*. [9, 10] and Brenner *et al*. [11] have shown that angiotensin-converting enzyme inhibitor (ACEi) and angiotensin receptor blockers (ARBs) reduce the progression of renal disease [12–16]. Data from the HOPE, LIFE and ALLHAT studies have confirmed the benefit of ACEi use in mild CKD [estimated glomerular filtration rate (eGFR) >60 mL/min/1.73m^2^ with proteinuria or structural defects] [17–20].

Few studies have included patients with advanced CKD at baseline. Moreover, it is difficult to dissociate the beneficial effect of ACEi from BP control. Indeed, in the HOPE sub-study using 24-h ambulatory BP in place of office BP, there was a significant reduction in BP from baseline in patients assigned to ramipril, which may have been an important mediator of benefit [18]. Ruggenenti *et al.* in a *post hoc* analysis of 322 patients suggested that therapy should be offered to all patients with CKD, even those with a GFR between 10 and 30 mL/min/1.73 m^2^ [21]. In this seminal study, there was no ‘nephroprotective’ effect when baseline proteinuria was <1.5 g/24 h, suggesting that the beneficial effects of ACEi may be limited to those with ‘pure’ glomerular disease rather than those with low-level proteinuria who may have ischaemic CKD. A Cochrane review of 49 studies containing 12 067 diabetic patients at all stages of CKD found that ACEi and ARBs improved hard end point [end-stage renal disease (ESRD)] and other outcomes, and appeared to reduce mortality (relative risk 0.78; 95% CI 0.61–0.98) [22]. The authors, however, cautioned against the conclusion that ACEi prevents progression of CKD, suggesting that any initial benefit may be due to their anti-proteinuric effects (probably reflecting better overall BP control), that there was little robust evidence of benefit in advanced CKD and that conclusions were based mainly on composite end points [22].

The rigor of some of these studies, which failed to dissociate renoprotective from antihypertensive effects of ACEi/ARBs [18], is now being questioned. Renoprotection from ACEi/ARB may be lost in more advanced disease where significant ischaemic nephropathy is present. This hypothesis is supported by reports in diabetic and non-diabetic patients with CKD indicating that ACEi/ARBs may accelerate renal progression, in spite of a beneficial anti-proteinuric effect [23–25]. Combined ACEi/ARB treatment has been shown to worsen renal outcomes in patients at high cardiovascular risk and increases the risk of hyperkalaemia and acute kidney injury [26–28]. The Telmisartan Randomized Assessment Study in ACE intolerant subjects with cardiovascular disease (TRANSCEND) demonstrated both a greater decline in eGFR and greater incidence of doubling of serum creatinine on Telmisartan (HR 1.59; 95% CI 1.04–2.41) [29].

In a recent observational study, withdrawal of ACEi/ARB therapy in 52 older patients with advanced CKD led to a mean increase in eGFR of 10 mL/min/1.73 m^2^ over 12 months and an increase or stabilization in eGFR in all but four patients. There was a small increase in BP but no change in risk of cardiovascular events [30]. There was also an association between the increase in BP levels upon discontinuation of ACEi/ARB and improved renal function [31]. The close interaction of the kidney and the heart is critical to survival, and the risk factors for poor cardiovascular outcomes in the general population and in early CKD are associated with better outcomes in advanced CKD [32, 33]. There are no studies assessing the benefits of ACEi/ARB therapy in cardiovascular risk reduction in advanced non-dialysis CKD. Indeed, although lowering BP reduces cardiovascular events, evidence suggests that ACEi/ARBs are not superior to other antihypertensives in this regard [34, 35]. Several randomized controlled studies in dialysis patients have shown increased cardiovascular events with the use of ACEi [36–38]. Indeed, the rate of decline of renal function remains a strong predictor of mortality [39–41].

## RESEARCH QUESTION

Does a strategy of discontinuing ACEi, ARBs or their combination in patients with advanced (Stage 4 or 5) progressive CKD lead to stabilization or improvement in renal function over a 3-year follow-up period, provided that good BP control is maintained with other agents, compared with a strategy of continuing ACEi and/or ARB?

## MATERIALS AND METHODS

### Study design

**FIGURE 1:**
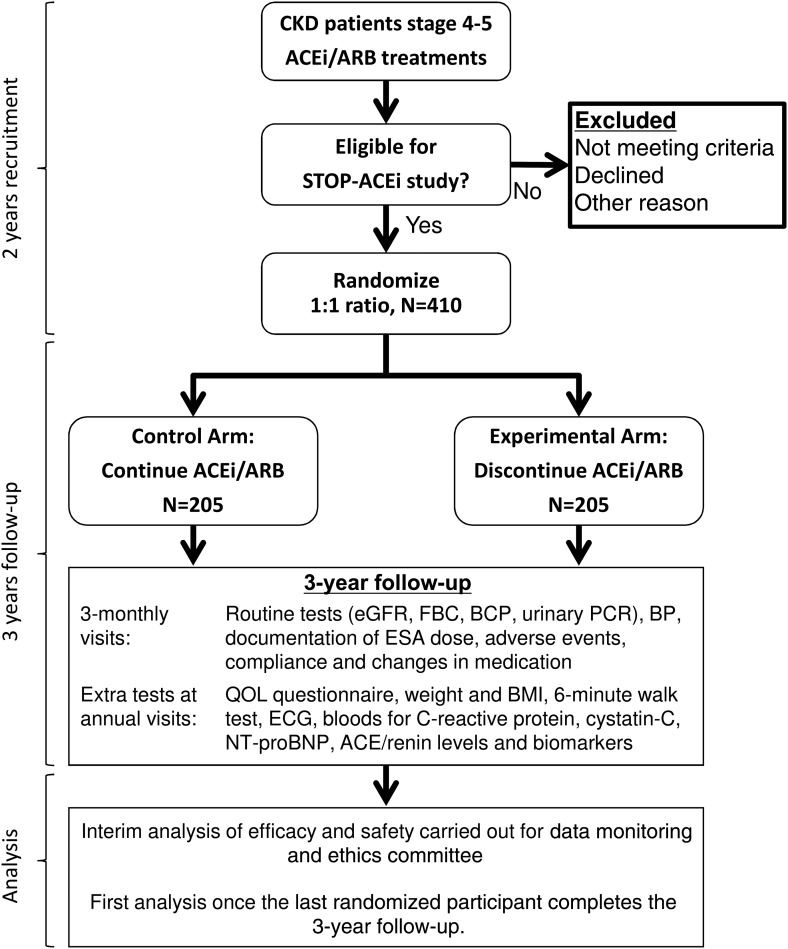
Schema for STOP-ACEi trial. BCP, biochemical profile (including urea and electrolytes and liver function tests); BMI, body mass index; FBC, full blood count; QOL, quality of life.

STOP-ACEi (trial registration: current controlled trials, ISRCTN62869767) is an investigator-led, multicentre, open-label, randomized controlled clinical trial of 410 participants aged 18 years or over with advanced (Stage 4 or 5) progressive CKD receiving ACEi, ARBs or both. Patients will be followed up 3 monthly for 3 years (Figure 1).

### Recruitment

Participants will be recruited from at least 15 UK renal units. Site staff will screen for potential eligible participants using the inclusion/exclusion criteria. If the participant is interested in taking part, the trial will be discussed with them in detail, and they will have the opportunity to ask questions. Participant eligibility will be confirmed with regard to the inclusion and exclusion criteria, and informed consent will be sought.

### Inclusion and exclusion criteria

Inclusion criteria are as follows:

- Aged ≥18 years (male or female)
- CKD Stage 4 or 5 [eGFR <30 mL/min/1.73 m^2^ using the modification of diet in renal disease (MDRD) equation] and must not have received a kidney transplant or be on dialysis
- Progressive deterioration in renal function (fall in an eGFR of >2 mL/min/year over previous 24 months) as measured by linear regression analysis based on a minimum of three eGFR measurements, with at least one reading from the previous 3 months
- Treatment with either an ACEi or ARB or a combination of both for >6 months with at least 25% of the maximum recommended daily dose
- Resting BP ≤160/90 mmHg when measured in accordance with British Hypertension Society guidelines in clinic or recent home BP reading within the previous month or a 24-h ambulatory BP measurement within the last 3 months
- At least 3 months of specialist renal follow-up
- Written, signed informed consent to the trial

Exclusion criteria are as follows:

- Aged <18 years
- Uncontrolled hypertension (>160/90 mmHg) or requirement for five or more agents to control BP
- Undergoing dialysis therapy
- Previous kidney transplant
- Any condition that, in the opinion of the investigator, makes the participant unsuitable for trial entry due to prognosis/terminal illness with a projected survival of <12 months
- History of myocardial infarction or stroke in preceding 3 months
- Participation in an interventional research study in preceding 6 weeks
- Pregnancy confirmed by positive pregnancy test or breastfeeding
- Inability to provide informed consent (e.g. due to cognitive impairment)
- Immune-mediated renal disease requiring disease-specific treatment
- Known drug or alcohol abuse
- Inability to comply with the trial schedule and follow-up

### Study enrolment and randomization

Following informed consent and completion of the baseline assessments, participants will be randomized into the trial in a 1∶1 ratio to either continue with their ACEi and/or ARB treatment (control arm) or to discontinue their ACEi and/or ARB treatment (experimental arm). Randomization will be provided by a computer-generated programme at the Clinical Trials Unit, using a minimization algorithm to ensure balance between the arms with regard to important clinical variables. The minimization variables will be diabetes (Type I diabetes, Type II diabetes or non-diabetic), BP {mean arterial pressure [MAP] measured as [(2 × diastolic) + systolic]/3; <100 or ≥100}, age (<65 or ≥ 65 years), proteinuria (protein:creatinine ratio (PCR) <100 or ≥100) and eGFR (<15 or ≥ 15 mL/min).

### Study treatment

#### Active treatment (experimental)

All ACEi and/or ARB treatment will be discontinued. In order to compensate for the loss of antihypertensive activity, additional antihypertensive treatment will be commenced to keep BP within recommended guidelines. Any antihypertensive agents used in routine clinical practice are permitted to control BP throughout the trial, but excluding ACEi or ARBs. The choice of antihypertensive will depend on other treatment being taken by the participant and will be at the discretion of the responsible clinician. Any antihypertensive agent used in routine clinical practice is permitted, but excluding ACEi and ARBs except as a last resort.

#### Control arm

Participants will continue on ‘standard’ care and will continue with their ACEi and/or ARB treatment. The choice and dose of ACEi and/or ARB will be at the discretion of the responsible clinician.

#### Both treatment groups

In both groups, BP will be controlled to the target pressure outlined in the National Institute for Health and Care Excellence (NICE) Hypertension guideline and NICE CKD guideline [42, 43] of ≤140/85 mmHg. The monitoring of BP will be as per the NICE CKD guideline.

#### Blinding

Trial treatment will be open label, due to the practical difficulties and costs associated with using placebo in a trial of drug withdrawal. However, the primary outcome measure is an objective laboratory measure that reduces the need for blinding. Additionally, investigators taking part in the trial will remain blind to all trial outcome data for the duration of the trial to minimize bias, with un-blinded data presented only to the independent Data Monitoring and Ethical Committee (DMEC).

### Assessments

Patients will be assessed at 3 monthly intervals from baseline to 3 years in the patient’s routine outpatient clinic visit as per the trial schedule of assessments (Table 1) [42].

**Table 1.** Schedule of assessments

Trial visit number	1	Phone call	2	3	4	5	6	7	8	9	10	11	12	13
Month	0		3	6	9	12	15	18	21	24	27	30	33	36
Check eligibility against inclusion/exclusion criteria, informed consent, randomization	✓													
Demographic and lifestyle data^a^	✓													
Medical history including cardiovascular comorbidities and CKD aetiology	✓													
Height	✓													
Weight and BMI	✓					✓				✓				✓
BP	✓		✓	✓	✓	✓	✓	✓	✓	✓	✓	✓	✓	✓
6 min walk test	✓					✓				✓				✓
KDQoL-SF™ v1.3 questionnaire	✓					✓				✓				✓
12-lead ECG	✓					✓				✓				✓
Record data from existing cardiac echocardiograms	✓		✓	✓	✓	✓	✓	✓	✓	✓	✓	✓	✓	✓
Record medication changes including ESA dose	✓	✓	✓	✓	✓	✓	✓	✓	✓	✓	✓	✓	✓	✓
Assess compliance with trial treatment allocation		✓	✓	✓	✓	✓	✓	✓	✓	✓	✓	✓	✓	✓
Adverse event documentation		✓	✓	✓	✓	✓	✓	✓	✓	✓	✓	✓	✓	✓
Lab assessments	✓		✓	✓	✓	✓	✓	✓	✓	✓	✓	✓	✓	✓
Routine tests (performed locally)^b^	✓					✓				✓				✓
C-Reactive Protein (performed locally)	✓					✓				✓				✓
Additional trial tests (analysed centrally)^c^	✓					✓								✓
Serum and urine samples taken for optional biomarker analysis														

^a^To include date of birth, gender, ethnicity, smoking status and alcohol intake.

^b^To include creatinine, eGFR, haemoglobin, mean cell volume, mean corpuscular haemoglobin, platelets, sodium, potassium, bicarbonate, calcium, phosphate, alkaline phosphatase, albumin, total protein, alanine transferase and urinary protein:creatinine or albumin:creatinine ratio.

^c^To include cystatin-C, NT-proBNP and ACE/renin levels in a sample of participants.

Demographic data (e.g. date of birth, gender, smoking status, weight, height) and a detailed disease history (e.g. cardiovascular comorbidity, antihypertensive medications) will be collected at baseline. BP will be recorded at each visit, and changes to antihypertensive and other concomitant medications will be recorded. A 12-lead electrocardiogram (ECG), quality of life (using KDQOL-SF^TM^ v1.3) and physical function (using 6 min walk test) will be collected at baseline and then annually.

For the purpose of the trial, urine and blood samples will be taken at baseline and each 3 monthly assessment. These samples will be used for routine tests and additional tests as part of the trial (Table 1).

### Primary outcome measure

The primary outcome measure is difference in renal function measured using the MDRD four-variable eGFR at the 3 years of follow-up.

### Secondary outcome measures

#### Renal events

- The number of participants starting renal replacement therapy or sustaining a >50% decline in eGFR
- Time taken to reach ESRD or need for renal replacement therapy

#### Quality of life and physical function

- Participant quality of life and well-being (KDQOL-SF™ v1.3 questionnaire)
- Participant physical function (6 min walk test)

#### Clinical events

- Hospitalization rates from any cause
- BP

#### Laboratory measures

- Serum cystatin-C

#### Safety

- Safety of intervention related to potential harm (e.g. increased cardiovascular events such as heart failure, myocardial infarction, stroke or death, participant survival in each group)

### Mechanistic outcome measures

- Urine protein excretion
- Blood haemoglobin concentration
- Dose of erythropoietin stimulating agent (ESA) administered

### Statistical considerations

#### Sample size

An observational study provided data on eGFR in 52 patients with advanced CKD in the 12 months prior to stopping ACEi/ARB treatment, at the point of stopping ACEi/ARB and 12 months after stopping (Table 2) [31]. These data form the basis of the sample size calculation.

**Table 2.** Data that formed the basis of the sample size calculation

Time point relative to stopping ACEi/ARB	eGFR (mL/min/1.73 m^2^), mean ± SE (SD)
−12 months	22.9 ± 1.4 (10.1)
0	16.38 ± 1 (7.2)
+12 months	26.6 ± 2.2 (15.9)

eGFR in patients with CKD who underwent ACEi/ARB withdrawal in a previously published study.

To err on the side of caution, the largest standard deviation was used to estimate the variability in eGFR (i.e. SD of 16 mL/min/1.73 m^2^) for the sample size calculation. To detect a minimum relevant difference between groups of 5 mL/min/1.73 m^2^ (i.e. effect size of 0.31) with 80% power and alpha = 0.05 (using a two-sample *t*-test), a total of 410 participants (205 per group) will need to be recruited (this includes allowance for 20% dropout). Data from Beddhu *et al*. who used propensity scores in a multivariate model in Dialysis Morbidity and Mortality Study Wave 2 patients showed that each 5-mL/min fall in MDRD GFR was associated with an increased hazard of death in a multivariable Cox model (HR 1.14; P = 0.002) [44].

#### Statistical analysis

The Statistical Analysis Plan will describe the planned analyses for STOP-ACEi in full detail. A summary of the main analyses are given here. All analysis will be based on the intention to treat principle, and a P-value of <0.05 will be considered statistically significant. The primary outcome is the continuous measure eGFR at 3 years. These data will be summarized using means and standard deviations, with differences in means and 95% CIs reported. The two groups will be compared at 3 years using both a two-sample *t*-test and ANCOVA to adjust for baseline values. Longitudinal plots of the data over time will be constructed for visual presentation of the data. As a secondary analysis, a repeated measures analysis, including a treatment by time cross-term, will be carried out on all data across the 3 years of follow-up.

Continuous secondary outcome measures (e.g. BP, quality of life) will be analysed in the same way as the primary outcome. Categorical (dichotomous) outcome measures (e.g. hospitalization rates) will compare the proportion of participants and percentages using a χ^2^ test, with relative risks and 95% CIs reported. Time-to-event outcomes (e.g. time to ESRD, mortality) will be analysed using survival analysis and log-rank methods. Kaplan–Meier survival curves will be constructed for visual presentation of time-to-event comparisons. Treatment effects will be expressed as HRs with 95% CIs.

Several *a priori* subgroup analyses with respect to the minimization variables for both primary and secondary outcomes will be performed. These analyses will be considered hypothesis generating.

## MONITORING

### Safety reporting

It is expected that the risk of the trial intervention, withdrawal of ACEi and/or ARB, will not be significantly higher than that of standard medical practice. The incidence of adverse events, including cardiovascular events, will be closely monitored by the trial oversight committees. All adverse events will be reportable to the STOP-ACEi trial office until each participant's final assessment at 3 years.

### Trial steering committee

An independent Trial Steering Committee will provide oversight of the study. The independent members of this committee are Dr Richard Haynes (Consultant Nephrologist; chair), Dr Nick Selby (Consultant Nephrologist) and Christopher Allison (Patient Representative).

### Data monitoring committee

A DMEC will independently monitor the efficacy and safety analysis reports at least annually. The independent members of this committee are Dr John Firth (Consultant Nephrologist; chair), Dr Paul Kalra (Consultant Cardiologist) and Mrs Merryn Voysey (Statistician).

## REGULATORY ASPECTS

The RCT will be conducted according to the standards of the International Conference on Harmonisation-Good Clinical Practice (GCP) and the Research Governance Framework for Health and Social Care. Pharmaco-vigilance reporting will comply with the Medicines for Human Use (Clinical Trials) Regulations 2004 and Amended Regulations 2006. Written informed consent will be provided by all patients prior to randomization and any study-related procedures.

STOP-ACEi is sponsored by the Hull and East Yorkshire Hospitals NHS Trust (R1578). The Medicine and Healthcare Products Regulatory Authority clinical trial authorization reference is 21411/0242/001-0001. The EudraCT number is 2013-003798-82.

## ETHICAL COMMITTEE APPROVAL

Ethical approval for STOP-ACEi was granted by the National Research Ethics Service Committee Yorkshire and the Humber—Leeds East (13/YH/0394) on 29 January 2014.

## DISCUSSION

Studies on the effects of ACEi/ARB in advanced CKD are lacking. In addition, published studies have failed to dissociate the putative renoprotective effects that are specific for ACEi/ARBs from their antihypertensive effect. Also, the benefits of the association between reducing protein excretion and the doubling of serum creatinine have recently been challenged by clinical trials such as ACCOMPLISH and ONTARGET, where a reduction in proteinuria/albuminuria was associated with accelerated CKD decline. The results of STOP-ACEi will provide evidence on whether discontinuation of ACEi/ARB is beneficial to renal function (improvement/stabilization) and improves other important parameters including laboratory (hyperkalaemia, anaemia) and clinical outcomes (hospitalization rates, physical function and quality of life) without causing an increase in cardiovascular events. It aims to clarify whether the benefits of this intervention outweigh the risks. It is, therefore, hoped that this pivotal study can provide new findings to allow future consideration of a large randomized controlled trial with mortality outcomes in this important group of patients.

### Trial status

STOP-ACEi opened to recruitment on 2 July 2014, and the first patient was recruited on 11 July 2014. As of 11 Sep 2015, 118 patients (29%) were recruited into the study.

## CONFLICT OF INTEREST STATEMENT

None declared.

(See related article by Solbu and Jardine. ‘To block or not to block’; whether to continue renin–angiotensin–aldosterone system blockade in advanced chronic kidney disease. *Nephrol Dial Transplant* 2016; 31: 171–173.)
